# New synonyms of two Arabian ants of the genus *Monomorium* Mayr, 1855 (Hymenoptera, Formicidae)

**DOI:** 10.3897/zookeys.505.9441

**Published:** 2015-05-21

**Authors:** Mostafa R. Sharaf, Cedric A. Collingwood, Hathal M. Al Dhafer, Mohammed S. Al mutairi, Abdulrahman S. Aldawood

**Affiliations:** 1 Plant Protection Department, College of Food and Agriculture Sciences, King Saud University, Riyadh 11451, P. O. Box 2460, Saudi Arabia; 2 18 Milton Street, Skipton, North Yorkshire, BD23 2ED, U. K.; 3 Saudi Wildlife Authority, Riyadh 11575, P.O. Box 61681, Saudi Arabia.

**Keywords:** Arabian Peninsula, Middle East, Saudi Arabia, United Arab Emirates, synonymy, taxonomy, new designation

## Abstract

Synonymy of two Arabian *Monomorium* Mayr, 1855 species is proposed: *Monomorium exiguum* Forel, 1894 = *Monomorium desertorum* Collingwood & Agosti, 1996, **syn. n.**; *Monomorium subopacum* Smith, 1858 = *Monomorium mintiribe* Collingwood & Agosti, 1996, **syn. n.** A lectotype for *Monomorium venustum* Smith, 1858 is designated. Information on nesting habits of *Monomorium exiguum* and *Monomorium venustum* in the Kingdom of Saudi Arabia are provided for the first time. Recently collected records for *Monomorium exiguum*, *Monomorium subopacum*, and *Monomorium venustum* from the Kingdom of Saudi Arabia and United Arab Emirates are listed.

## Introduction

The first published work on the ant genus *Monomorium* Mayr, 1855 for the Kingdom of Saudi Arabia (KSA) was by Collingwood (1985), who listed and keyed 20 species from the country. The genus was subsequently treated comprehensively for the Arabian Peninsula by Collingwood and Agosti (1996). The authors reported 53 species, 32 of which were described as new, (including 15 from the KSA, 10 from Oman, five from Yemen, and two from Kuwait). Collingwood et al. (2011) treated the myrmecofauna of the United Arab Emirates (UAE) and reported 29 *Monomorium* species. Three species were recorded from Socotra Island (Collingwood et al. 2004) and a new species, *Monomorium nimihil* Collingwood, 2004 was described.

Recently the *Monomorium* fauna of KSA has received renewed attention, with the first record of *Monomorium exiguum* Forel, 1894 (Aldawood and Sharaf 2009) and descriptions of three new species, *Monomorium moathi* Sharaf & Collingwood, 2010 (Aldawood et al. 2010); *Monomorium dryhimi* Aldawood & Sharaf, 2011 (Aldawood and Sharaf 2011) and *Monomorium sarawatensis* Sharaf & Aldawood, 2013 (in El-Hawagry et al. 2013). During two visits to the World Museum, Liverpool, United Kingdom, two new synonyms were discovered for Arabian *Monomorium*.

## Materials and methods

### Abbreviations of museums

BMNH The Natural History Museum, London, United Kingdom.

MHNG Museum d’Histoire Naturelle, Geneva, Switzerland.

NHMB Naturhistorisches Museum, Basel, Switzerland.

WMLC World Museum Liverpool, Liverpool, United Kingdom.

The numbers between parentheses in material examined refer to individual workers.

## Results and discussion

### Monomorium exiguum

Taxon classificationAnimaliaHymenopteraFormicidae

Forel, 1894

Figs 1–4

Monomorium exiguum For full synonymy see Heterick (2006), pp. 115–116.
Monomorium exiguum Forel, 1894: 85. (lectotype worker) Ethiopia. Afrotropic. “Ethiopia, Sudabessinien.” (MHNG), http://www.antweb.org/specimen/CASENT0101870 [Image of type specimen examined].
Monomorium desertorum Collingwood & Agosti, 1996: 344 (w.) Saudi Arabia. Afrotropic.” (WMLC), http://www.antweb.org/specimen/CASENT0906343. Syntype worker [examined], Syn. n.

#### Material examined.

Saudi Arabia, Baha, Dhi Ayn Archeological Village, 18.v.2010, 20.132°, 41.004°, 741m, (M. R. Sharaf, leg.) (21); Saudi Arabia, Riyadh, Oyaina, 28.iv.2010; 25.011°, 46.493°, 749m, (M. R. Sharaf, leg.) (3); Saudi Arabia, Riyadh, Qarina Valley, 5.xi.2009, 25.273°, 46.289°, 761m, (M. R. Sharaf, leg.) (3); Saudi Arabia, Baha, Dhi Ayn Archeological Village, 20.ix.2011, 20.132°, 41.004°, 744m, (M. R. Sharaf, leg.) (10); Saudi Arabia, Almajardah, wadi Khat, 10.xi.2012, 19.001°, 41.016°, 513m, (M. R. Sharaf, leg.) (6); Saudi Arabia, wadi Shahdan (Jizan), 13.xi.2012, 17.472°, 42.856°, 200m, (M. R. Sharaf, leg.) (8); Saudi Arabia, Wadi Aljora near Abadan, 12.xi.2012, 17.005°, 43.001°, 465m, (M. R. Sharaf, leg.) (6); Saudi Arabia, Baha, Wadi Elzaraeb, 9.v.2011, 20.073°, 41.387°, 2086m, (M. R. Sharaf, leg.) (1); Saudi Arabia, Abu Arish, 10.iv.2012, 17.013°, 42.802°, 90m, (M. R. Sharaf, leg.) (6); Saudi Arabia, Dhi Ayn Archeological Village, 11.v.2011, 19.929°, 41.442°, 741m, (M. R. Sharaf, leg.) (3); Saudi Arabia, Baha, Dhi Ayn Archeological Village, 7.iv.2013, 19.929°, 41.442°, 744m, (M. R. Sharaf, leg.) (4); Saudi Arabia, AlUrdiyah gov., Wadi Gonouna, 12.v.2011, 19.429°, 41.605°, 353m, (M. R. Sharaf, leg.) (20); Saudi Arabia, Al Bahah, Wadi Turabah, AlMandaq, 14.v.2011, 20.211°, 41.288°, 1793m, (M. R. Sharaf, leg.) (7); Saudi Arabia, Dhi Ayn Archeological Village, 15.v.2011, 19.929°, 41.442°, 741m, (M. R. Sharaf, leg.) (1); Saudi Arabia, Al Bahah, Wadi Turabah, AlMandaq, 10.v.2011, 20.211°, 41.288°, 1793m, (M. R. Sharaf, leg.) (1); Saudi Arabia, Riyadh, Hawtet Bani Tamim, 20.i.2014, 23.480°, 46.844°, 597m, (M. R. Sharaf, leg.) (3); Saudi Arabia, Al Qatif, El Naft, 23.iii.2012, 26.510°, 49.969°, 30m, (M. R. Sharaf, leg.) (2); UAE, Khor al-Khwair, 25.57.56.03, 8.iii.2007, (M. Hauser et al.) (1); UAE, Sharjah, 25.21.55.24, 28.ii–12.iv.2011, (M. Hauser et al.) (1); UAE, Wadi Bih dam, 25.48.56.04, 16–31.xii.2009, (M. Hauser et al.) (1).

**Figures 1–4.**
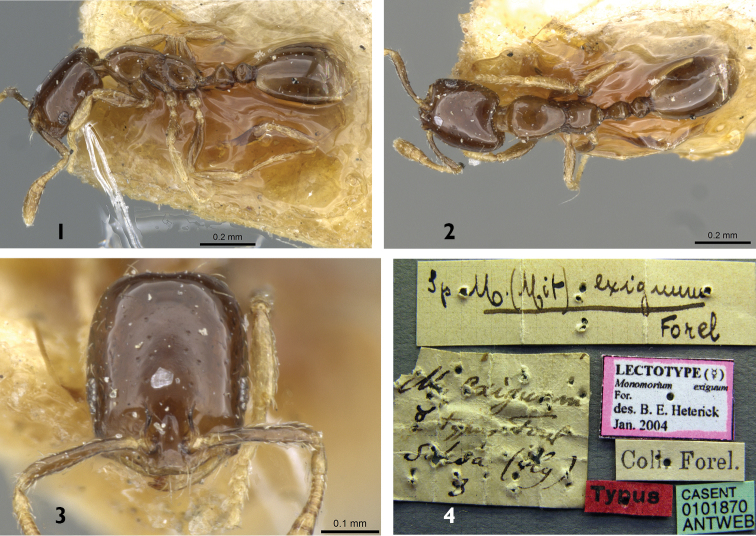
*Monomorium exiguum* (worker), CASENT0101870. **1** Body in profile **2** Body in dorsal view **3** Head in full-face view **4** specimen label. Photo Zach Lieberman, http://antweb.org/

#### Remarks.

Only a single paratype specimen with the same data as the holotype exists at WMLC. The holotype and other paratypes are considered lost.

The description of *Monomorium desertorum* in Collingwood and Agosti’s (1996) indicated that the eyes are located anterior to the midlength of head, the scapes when retracted back do not reach the posterior margin of head, the antennae are 11 segmented, and the body is not sculptured except for the metanotal cross-ribs. Comparison was made of the single available paraype worker of *Monomorium desertorum* with the lectotype worker of *Monomorium exiguum* was carried out. We here treat *Monomorium desertorum* as a junior subjective synonym of *Monomorium exiguum*.

#### Habitat.

The vast majority of *Monomorium exiguum* nests that were collected in KSA were found to be associated with leaf litter and topsoil layers where workers foraged. Frequently nests were directly in the soil. The nesting habits of *Monomorium exiguum* however, are diverse. In a site located in the southwestern mountains of the KSA, the species was found nesting in loose sandy soil with high moisture content and among roots of small *Portulaca oleracea* L. (Portulacaceae) plants beneath a date palm tree, *Phoenix dactylifera* L. (Arecaceae). Several worker series were nesting in a humid clay soil under banana trees. Other worker series were collected under a rock next to *Juniperus procera* Hochst. ex Endlicher (Cupressaceae) and *Acacia* spp. (Mimosaceae) trees. Another nest was found in thick layer of leaf litter under a large and old *Ficus benghalensis* L. (Moraceae) tree where the soil was rich in decayed organic matter. Some nests were found in leaf litter under *Calotropis procera* (Aiton) W.T.Aiton (Asclepiadaceae )and next to a mango tree (*Mangifera* sp., Anacardiaceae).

### Monomorium subopacum

Taxon classificationAnimaliaHymenopteraFormicidae

(Smith, 1858)

Figs 5–8

Monomorium subopacum For full synonymy, see Heterick (2006), p. 103.
Myrmica subopaca Smith, 1858: 127 (w.q.) (paralectotype worker, designated by B. E. Heterick, September, 2004) Portugal (Madeira Is.). Afrotropic. “Portugal (Madeira Island), coll. T.V. Wollaston. (BMNH), http://www.antweb.org/specimen/CASENT0010949 [Image of type specimen examined].
Monomorium mintiribe Collingwood & Agosti, 1996: 350, fig. 23 (w.q.m.) Oman. Palearctic. Bilad Ban. 17.i.1986, coll. W. Buttiker. (WMLC), Paratype worker [examined]. Syn. n.

#### Material examined.

UAE, Ar-Rafah, 25.43.55.52, 1–8.iii.2011, (M. Hauser et al.) (1); UAE, Ar-Rafah, 25.43.55.52, 1.ii–31.iii.2010, (M. Hauser et al.) (1); UAE, Ar-Rafah, 25.18.56.07, 22.vi–2.vii.2010, (M. Hauser et al.) (1); UAE, Jebel Jibir, 25.39.56.07, 11–13.iv.2011, (M. Hauser et al.) (1).

**Figures 5–8.**
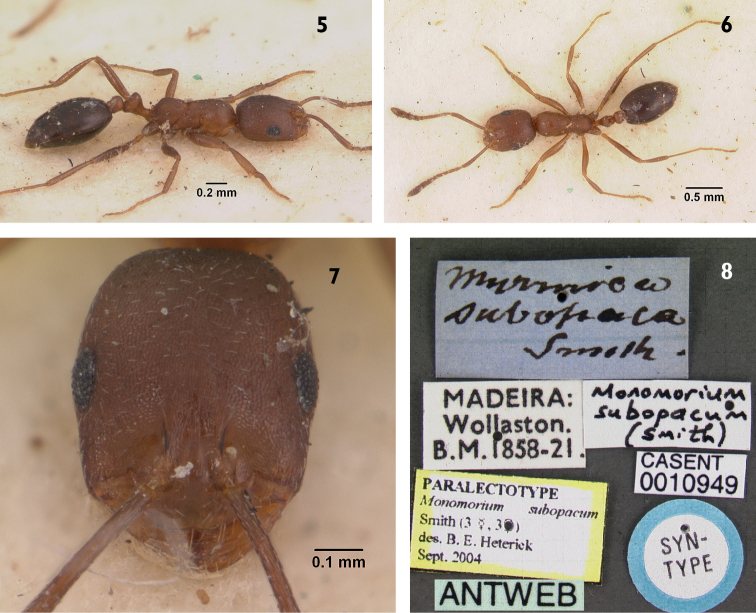
*Monomorium subopacum* (paralectotype worker), CASENT0010949. **5** Body in profile **6** Body in dorsal view **7** Head in full-face view **8** specimen label Photo April Nobile, http://antweb.org/

#### Remarks.

The holotype and 10 paratypes of *Monomorium mintiribe* seem to be lost. Extensive searches at both WMLC and NHMB failed to locate type material except for a single paratype specimen labeled in red at the WMLC. In addition, despite the fact that the label information for the paratype specimen in WMLC does not exactly match the information in Collingwood and Agosti (1996), we consider this specimen as part of the original type series. Collingwood and Agosti (1996) indicated the following paratypes: 1 male, 2 queens, 2 workers, “Oman, Bilad Bani, 20°03'N, 59°17'E, coll. W. Buttiker”, whereas the data on the paratype specimen in WMLC is “Bilad Ban, Oman, W. Buttiker, 17.i.1986.” The second author (C. A. Collingwood) confirms that the single remaining specimen is an originally designated paratype. The original description of *Monomorium mintiribe* did not compare this taxon with related congeners. The single paratype is identical to the paralectotypes of *Monomorium subopacum* and the original description agrees with this. Therefore, *Monomorium mintiribe* is treated here as a junior subjective synonym of *Monomorium subopacum*.

### Monomorium venustum

Taxon classificationAnimaliaHymenopteraFormicidae

(Smith, 1858)

Figs 9–12

Myrmica venusta Smith, 1858: 126 (w.) (lectotype worker) Syria. Palaearctic. (BMNH “E” 1015257) [**new designation**].

#### Material examined.

Saudi Arabia, Al Atawla (Baha-Taif RD), Wadi Bawah, 8.xi.2012, 21.004°, 41.247°, 1310m, (M. R. Sharaf, leg.) (10); Saudi Arabia, Baha, Wadi Elzaraeb, 9.v.2011, 20.073°, 41.387°, 2086m, (M. R. Sharaf, leg.) (3); Saudi Arabia, Riyadh, Dirad, 30.xii.2009, 24.409°, 46.662°, 588m, (M. R. Sharaf, leg.) (6); Saudi Arabia, Al Bahah, Wadi Turabah, AlMandaq, 19.ix.2011, 20.242°, 41.262°, 1751m, (M. R. Sharaf, leg.) (6); Saudi Arabia, Riyadh, Alhota, 19.iv.2008, (M. R. Sharaf, leg.) (7); Saudi Arabia, Riyadh, Wadi Hanifa, 11.iv.2013, 24.671°, 46.595°, 641 (M. R. Sharaf, leg.) (14); Saudi Arabia, Al Bahah, Wadi Turabah, AlMandaq, 10.v.2011, 20.211°, 41.288°, 1751m, (M. R. Sharaf, leg.) (4).

**Figures 9–12.**
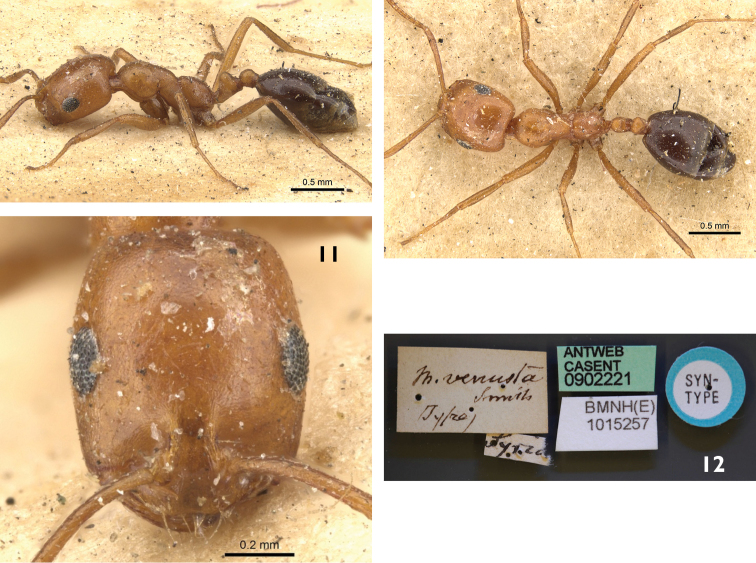
*Monomorium venustum* (Lectotype worker), CASENT0902221. **9** Body in profile **10** Body in dorsal view **11** Head in full-face view **12** specimen label. Photo Will Ericson, http://antweb.org/

#### Remarks.

Originally, *Monomorium venustum* was described based on syntypes of the worker caste from Syria. Here we designate a lectotype with the following data, “*Myrmica venusta* Smith, type, BMNH (E), 1015257”. The Lectotype is deposited at BMNH.

#### Habitat.

Workers of *Monomorium venustum* build nests directly into the ground under stones and rocks, directly into the ground. This species apparently prefers to nest in soil with high moisture content as observed in many locations in KSA. In the southwestern mountains of the KSA, nests were constructed next to *Juniperus procera* Hochst. ex Endlicher (Cupressaceae) and *Acacia* spp. (Mimosaceae) trees. In addition, the species is usually foraging in areas with dense green flowering grasses that covering the ground. A single nest was found existing next to *Mentha longifolia* (L.) Huds. (Lamiaceae). Myrmecophilous arthropods (e.g. small beetles, isopods and millipedes) were found inside some nests.

## Supplementary Material

XML Treatment for Monomorium exiguum

XML Treatment for Monomorium subopacum

XML Treatment for Monomorium venustum
